# Dysphagia and its impact on quality of life in rare neuromuscular disorders

**DOI:** 10.1055/s-0045-1804920

**Published:** 2025-03-19

**Authors:** Déborah Santos Sales, Mariana Beiral Hammerle, Vívian Pinto de Almeida, Clarissa de Araujo Davico, Patricia Gomes Pinheiro, Rayanne da Silva Souza, Stephanie de Freitas Canelhas, Marcele Silva Carvalho, Karina Lebeis Peres

**Affiliations:** 1 Universidade Federal do Estado do Rio de Janeiro, Rio de Janeiro RJ, Brazil.; 2 Universidade Federal do Estado do Rio de Janeiro, Hospital Universitário Gaffrée e Guinle, Rio de Janeiro RJ, Brazil.; 3 Casa Hunter, São Paulo SP, Brazil.; 4 Universidade Veiga de Almeida, Rio de Janeiro RJ, Brazil.

**Keywords:** Deglutition Disorders, Neuromuscular Diseases, Amyotrophic Lateral Sclerosis

## Abstract

**Background** Patients with neuromuscular diseases (NMDs) often face swallowing difficulties (dysphagia) as part of their condition.

**Objective** To determine the prevalence of self-reported swallowing disorders in patients with rare NMDs and examine their correlation with related quality of life (QoL).

**Methods** The study included 103 patients with confirmed rare NMDs. Dysphagia risk was assessed using the validated Eating Assessment Tool-10 (EAT-10), and QoL related to swallowing was measured with the SWAL-QoL survey. Correlations between EAT-10 and SWAL-QoL scores were analyzed. Additionally, the mean questionnaire scores were compared among patients classified as dysphagic, dysphagic with high aspiration risk, and nondysphagic.

**Results** The estimated prevalence of dysphagia in the cohort, based on EAT-10, was 52.4%. Higher scores were significantly correlated with poorer swallowing-related QoL, except for the sleep domain. The most affected SWAL-QoL domains were burden, eating desire, eating duration, food selection, communication, fear, mental health, social functioning, and dysphagia battery score (DBS), with significant differences observed among the classifications ( *p* < 0.001 for most domains, and *p* = 0.015 for eating desire). No statistically significant difference in swallowing QoL was found between sitters and walkers.

**Conclusion** Dysphagia is a prevalent symptom in patients with rare NMDs, affecting 52.4% of the cohort and significantly impacting QoL in nearly all domains except sleep.

## INTRODUCTION

Patients with neuromuscular diseases (NMDs) often face swallowing difficulties (dysphagia) as part of their condition. The most prevalent diseases, including Duchenne muscular dystrophy, myotonic dystrophy type 1, and amyotrophic lateral sclerosis (ALS), frequently involve dysphagia due to muscle weakness. 1 In adult NMD patients, dysphagia affects 34.9 to 80%, depending on various factors such as genetic mutation, symptoms, age of onset, progression rate, and prognosis. 2 3 4 5

Dysphagia can lead to serious functional impairments like aspiration pneumonia, malnutrition, and dehydration, increasing the risk of morbidity and mortality, as well as significantly impacting quality of life (QoL). 6 Specific conditions, like bulbar and progressive respiratory muscle weakness, are common in NMDs and can compromise safe and efficient swallowing. 4 Consequently, patients' social lives suffer, as these conditions' hinder participation in food-related events, like family dinners and other social gatherings, reducing their enjoyment of eating and overall QoL. This can result in higher rates of depression and social isolation among these patients. 3 7

A thorough assessment is essential for identifying individuals at risk of dysphagia to prevent and manage negative outcomes. Self-report tools are increasingly utilized in healthcare settings to identify patient-perceived symptoms, track symptom changes, and support a patient-centered care approach. 8 A recent study described current practices in the management of dysphagia in NMDs across Europe and found that the most frequent screening tools were the Eating Assessment Tool (EAT-10) and the Quality of Life in Swallowing Disorders (SWAL-QoL) with 32% and 28%, respectively. 9

The World Health Organization defines QoL as linked to an individual's ability to perform desired activities and their expectations, concerns, self-care, lifestyle, environment, as well as emotional, social, and professional satisfaction. It encompasses the individual's overall perspective on their wellbeing, not just health. 10

The SWAL-QoL questionnaire was designed to evaluate and measure QoL related to swallowing. 11 Numerous studies using SWAL-QoL have been published recently; 12 13 14 15 however, few have explored the correlation between self-reported swallowing issues and QoL in patients with rare NMDs.

Understanding QoL factors in NMDs is crucial for comprehensive patient care. Self-perception tests have proven to be significant in identifying patients at risk for dysphagia, necessitating more thorough assessments of oropharyngeal swallowing. 4

This study aims to determine the prevalence of self-reported swallowing disorders in patients with rare NMDs and correlate these with the related QoL in these individuals.

## METHODS

### Participants

We conducted a prospective, observational study from February 2021 to December 2023. A total of 135 patients were consecutively recruited from a specific outpatient clinic for neuromuscular disease that takes place weekly in a university hospital in their regular follow-up visits. All patients included in the study were diagnosed in our reference center specialized in rare NMDs and had onset of symptoms for a maximum of 6 months.

There were 32 patients excluded from the study due to insufficient data. The eligibility criteria included:

- A confirmed diagnosis of any neuromuscular disease (NMD);
- Age 18 years or older;
- Presence or absence of swallowing disorder complaints, when asked by the healthcare professional;
- Ability to consume some form of oral intake; and
- No feeding tube.

Patients were classified as elderly if they were 60 years or older. Participants were divided into two groups: sitters and walkers. We considered as “sitters”, patients who have lost the ability to walk and move in wheelchairs, and “walkers” as those who can walk freely, or with the use of orthoses, such as canes, crutches, or walkers.

This study adhered to the ethical guidelines outlined in the Declaration of Helsinki. Approval was obtained from the institution's Ethical Review Board (protocol number 38103720.6.0000.5291). All participants provided written informed consent and received a copy of their signed forms.

### Procedures

#### *Demographic Data*

Demographic data, including age, disease duration, sex, and neuromuscular disease type, were collected for each participant at the beginning of the research evaluation. Patients were asked to report any swallowing difficulties before data collection, but all protocols were applied regardless of their responses.

#### *Assessment of Dysphagia*

The clinical assessment included a qualitative analysis of patients' food intake, focusing on texture modifications and any compensatory strategies they adopted.

#### *Eating Assessment Tool-10*

The risk of dysphagia was assessed using the validated EAT-10, originally developed by Belafsky et al. 16 This widely used tool evaluates the risk of dysphagia, functional health status, and QoL in clinical practice. It consists of ten questions, each rated from 0 to 4 on a scale. A new variable was created, identifying patients with scores of 3 or higher as at risk for dysphagia. 16 A cutoff score of 8 was used to indicate a higher risk of aspiration, according to Plowman et al's study. 17

#### *Quality of Life in Swallowing Disorders (SWAL-QoL)*

The SWAL-QoL survey assesses the impact of swallowing difficulties on health-related QoL in patients with oropharyngeal dysphagia. 16 It comprises 44 items divided into 11 QoL concepts, seven of which are dysphagia-related (burden, food selection, eating duration, eating desire, fear, communication, social functioning, and mental health), and three are general QoL concepts (communication, fatigue, and sleep). It also includes a 14-question frequency of dysphagia symptoms battery (DSB) used as a status index. Each domain score is calculated based on two or more questions, ranging from 0 (extremely impaired) to 100 (no impairment). Lower scores indicate worse QoL in the respective domain. Although the questionnaires were self-administered, researchers assisted some patients due to educational limitations, reading difficulties, vision problems, or lack of eyeglasses.

### Statistical analysis

Descriptive statistics were obtained for demographic and clinical variables. Due to the asymmetrical distribution of response variable frequencies, nonparametric tests were employed to compare variables between patients with and without dysphagia risk. Continuous variables are presented as medians and interquartile ranges (IQRs), defined as the range between 25 and 75%. Spearman's rank correlation assessed the association between EAT-10 and SWAL-QoL subscales, and between EAT-10 and age. Median questionnaire scores between patients at risk and not for dysphagia were compared using the Mann-Whitney U test. A significance level of *p* < 0.05 was set for the analysis. Statistical analyses were conducted using the IBM SPSS Statistics for Windows (IBM Corp., Armonk, NY, USA) software, version 22.0.

## RESULTS

The study included 103 patients with a mean age of 46.5 ± 16.4 years. Among them, sixty patients were women (58.3%). Table 1 details the demographic data, including sex, age, and reported diseases. Completing the EAT-10 took less than 2 minutes, while the SWAL-QoL took approximately 15 minutes for most patients. In our cohort, the estimated prevalence of dysphagia risk based on the EAT-10 was 52.4%.

**Table 1:** Sample characteristics

Category			Values
Patients age, mean (SD)			46.5 ± 16.4
Adult (n, %)			87.0 ± 84.5
Elderly (n, %)			16.0 ± 15.5
Sex (n, % female)			60.0 ± 58.3
**NMD (n, %)**	Amyotrophic lateral sclerosis		54 (52.4)
		Bulbar onset	15 (27.7)
		Spinal onset	34 (62.9)
		Familial onset	3 (9.3)
	Spinal muscle atrophy		23 (22.3)
		Type 2	8 (34.8)
		Type 3	14 (61.0)
		Type 4	1 (4.2)
	Pompe disease		10 (9.7)
	Limb–girdle muscular dystrophy		9 (8.7)
		Type 2A	3 (33.3)
		Type 2B	3 (33.3)
		Type 2D	3 (33.3)
	Myotonic dystrophy		7 (6.8)
	EAT-10 score (SD)		8.5 ± 10.4
		Sitters (n, %)	58 (56.3)
		Walkers (n, %)	45 (43.7)

Abbreviations: EAT-10, Eating Assessment Tool-10; NMD, neuromuscular disease; SD, standard deviation.

The mean EAT-10 score was 8.5 ± 10.4, ranging from 0.0 to 40.0. All participants were on a full oral diet. The most prevalent neurological rare disease was ALS (52.4%, N = 54). Additional demographic and clinical characteristics are presented in Table 1 .

Table 2 shows the EAT-10 values for each NMD.

**Table 2:** Median EAT-10 scores stratified by neuromuscular diseases

NMD	EAT-10, mean (SD)
Amyotrophic lateral sclerosis	12.0 ± 11.4
Limb–girdle muscular dystrophy	6.8 ± 10.2
Myotonic dystrophy	5.3 ± 4.9
Spinal muscle atrophy	4.8 ± 8.0
Pompe disease	1.6 ± 2.9

Abbreviations: EAT-10, Eating Assessment Tool-10; NMD, neuromuscular disease; SD, standard deviation.

Notably, ALS patients had a mean EAT-10 score of 12, indicating a high risk of dysphagia and aspiration. Conversely, Pompe disease patients had the lowest EAT-10 score, suggesting safer swallowing.

Based on Spearman's rank correlation, there was a significant negative correlation between EAT-10 and SWAL-QoL on almost all subscales, except for sleep (rs = -0.103; *p* = 0.30). A negative correlation indicated a higher risk of dysphagia and worse SWAL-QoL ( Table 3 ).

**Table 3:** SWAL-QoL subscale correlations with EAT-10

SWAL-QoL subscales	EAT-10	
	Spearman's rho (r _s_ )		
Burden	−0.578*	< 0.001*
Eating desire	−0.240*	0.015*
Eating duration	−0.448*	< 0.001*
Food selection	−0.460*	< 0.001*
Communication	−0.414*	< 0.001*
Fear	−0.450*	< 0.001*
Mental health	−0.520*	< 0.001*
Social functioning	−0.454*	< 0.001*
Sleep	−0.103	0.302
Fatigue	−0.223	0.024*
DBS	−0.444	< 0.001*

Abbreviations: SWAL-QoL, QoL in Swallowing Disorders; EAT-10, Eating Assessment Tool-10; DBS, dysphagia battery score.

Note: * *p* < 0.05; second column, Spearman's value; third column, *p* value related to statistical analysis.

Table 4 compares the means of the SWAL-QoL subscales among patients without dysphagia risk (group 1), those with moderate risk (group 2), and those with dysphagia and high aspiration risk (group 3). Group 3 had the lowest SWAL-QoL scores, indicating poorer quality of life-related to swallowing. There were significant differences in the burden, eating desire, eating duration, food selection, communication, fear, mental health, social functioning, and dysphagia battery score (DBS) subscales ( *p* < 0.015 for eating desire, 0.001 for the others). These findings suggest that patients with EAT-10 scores of 8 or higher experience worse swallowing-related QoL than other groups.

**Table 4:** Scores obtained in SWAL-QoL stratified in sections

SWAL-QoL subscale scores	No dysphagia (n = 28)	Dysphagia (n = 45)	Dysphagia with high risk for aspiration	*p* -value
Burden ^a^	88.1 ± 27.1	84.6 ± 29.4	45.2 ± 36.1	< 0.001*
Eating desire ^a^	84.1 ± 21.6	83.0 ± 27.9	65.1 ± 33.1	0.004*
Eating duration ^a^	76.5 ± 338	79.2 ± 29.5	44.9 ± 33.5	< 0.001*
Food selection ^a^	90.5 ± 24.1	84.6 ± 29.4	62.0 ± 35.5	< 0.001*
Communication ^a^	83.7 ± 26.5	79.3 ± 30.7	51.2 ± 38.6	< 0.001*
Fear ^a^	77.0 ± 29.3	68.8 ± 34.7	44.4 ± 34.9	< 0.001*
Mental health ^a^	94.4 ± 18.6	83.4 ± 33.5	63.3 ± 36.1	< 0.001*
Social functioning ^a^	89.9 ± 24.3	77.1 ± 33.0	59.8 ± 37.8	< 0.001*
Sleep ^a^	54.2 ± 30.1	57.7 ± 21.9	49.2 ± 28.9	0.578
Fatigue ^a^	55.6 ± 34.0	49.3 ± 34.4	37.7 ± 33.8	0.050
DBS	86.3 ± 18.2	79.4 ± 26.5	62.7 ± 27.7	< 0.001*

Abbreviations: SWAL-QoL, QoL in Swallowing Disorders; DBS, dysphagia battery score.

Notes: ^a^ Median (first to third quartiles), *p* -value of the ANOVA test based on Bonferroni for the quantitative variables; * *p* < 0.05.

The section on *sleep problems* , which includes issues sleeping through the night, showed no statistically significant differences ( *p* = 0.302) among patients. Sleep problems are part of a subdomain of the SWAL-QoL scale, which includes the following questions: “Do you have trouble sleeping?” “Do you sleep through the night?”

A comparison was conducted between the means of the SWAL-QoL subscales among sitters and walkers, revealing no statistically significant difference between the groups ( Table 5 ).

**Table 5:** Scores obtained in SWAL-QoL stratified in groups

SWAL-QoL subscale scores	Sitters (n = 28)	Walkers (n = 45)	*p* -value
Burden ^a^	70.4 ± 36.9	70.7 ± 37.8	0.975
Eating desire ^a^	74.4 ± 30.8	77.9 ± 27.3	0.545
Eating duration ^a^	65.8 ± 36.8	63.2 ± 36.3	0.716
Food selection ^a^	79.3 ± 36.6	77.7 ± 31.8	0.809
Communication ^a^	70.5 ± 35.2	69.9 ± 36.2	0.932
Fear ^a^	66.5 ± 37.6	60.3 ± 33.8	0.378
Mental health ^a^	78.3 ± 34.2	82.4 ± 30.2	0.514
Social functioning ^a^	77.3 ± 33.9	75.6 ± 34.6	0.804
Sleep ^a^	49.3 ± 29.8	55.2 ± 27.7	0.307
Fatigue ^a^	48.6 ± 35.3	47.0 ± 34.4	0.820
DBS	77.5 ± 27.7	75.1 ± 24.2	0.627

Abbreviations: SWAL-QoL, QoL in Swallowing Disorders; DBS, dysphagia battery score.

Notes: ^a^ Mean ± SD (first to third quartiles); *p* -value of the *t* test for independent samples.

## DISCUSSION

This study aimed to evaluate the QoL in swallowing among patients with NMDs. In our sample, domains such as burden, eating duration, food selection, communication, fear, mental health, social functioning, and dysphagia (symptom frequency) were notably affected in the SWAL-QoL assessment. These findings align with several studies in the literature. 4 12 15 17 18

For instance, in a 2017 study involving 113 patients with oculopharyngeal muscular dystrophy, worse scores were reported in burden, eating duration, and fatigue within the SWAL-QoL assessment. 19

Moreover, Plowman et al.'s research on ALS patients demonstrated that EAT-10 is an effective tool for identifying aspiration risk, particularly with a cutoff score of 8. 17 Their findings revealed significantly higher scores in aspirators compared to patients who swallowed safely. 4 17 In our study, given that ALS was the most prevalent disease (52.4%) and the mean EAT-10 score in this population was 12.0, it reinforces the risk of aspiration in these patients.

Regarding sleep problems, our study found no statistically significant differences between patients with and without dysphagia. This finding contrasts with anticipated results, as individuals with NMDs frequently experience generalized weakness, directly impacting sleep quality, 20 irrespective of dysphagia presence. Additionally, early indications of nighttime noninvasive ventilation in our sample may contribute to the absence of sleep problems observed in our results.

The SWAL-QoL's domains exhibited statistical significance when comparing groups with and without dysphagia, particularly the group of patients with dysphagia and high aspiration risk (EAT-10 ≥ 8), which directly correlated with swallowing difficulties. Domains such as burden and fear—encompassing frustration, isolation, fear of choking, and aspiration pneumonia—negatively impacted the QoL of patients with dysphagia.

Paris et al. also noted statistically significant changes between the burden and eating duration domains, consistent with previous studies. Additionally, they highlighted the difficulty of oral communication as significant, possibly associated with increased depressive disorder and impaired social life. 15

Unexpectedly, our study found no statistically significant difference between the sitters and walkers groups concerning swallowing-related QoL. This could be attributed to the sample size discrepancy, with a notably larger number of sitters than walkers. Further studies are warranted to explore this aspect comprehensively.

Finally, it is important to highlight that the management of dysphagia in patients with rare NMDs requires a comprehensive and multidisciplinary approach. 9 The involvement of neurologists, otolaryngologists, speech therapists, nutritionists, physiotherapists, and psychologists are essential to meet the complex needs of this cohort, 21 especially when planning integrated end-of-life care. 9 This collaborative approach ensures that all aspects of the patient's condition are addressed, leading to better outcomes and improved QoL. Our outpatient clinic has a multidisciplinary team, where all patients are not only evaluated but also receive longitudinal monitoring from the entire team.

### Limitations

While our study provides valuable insights into the QoL in swallowing among patients with NMDs, several limitations should be acknowledged.

- Sample Size and Selection Bias: The sample size might not be representative of the entire population of patients with NMDs. Moreover, recruitment from a single center, such as our University Hospital, may introduce selection bias and limit the findings' generalizability to other settings or populations.
- Cross-Sectional Design: The study's cross-sectional design limits our ability to establish causality or determine the long-term effects of dysphagia on QoL. Longitudinal studies would provide a more comprehensive understanding of this matter.
- Self-Reported Measures: The reliance on self-reported measures, such as the EAT-10 and SWAL-QoL surveys, introduces the potential for recall bias and subjective interpretation, which may affect the accuracy of the results.
- Confounding Factors: The presence of confounding variables, such as comorbidities, concomitant medications, and socioeconomic factors, could influence the relationship between dysphagia and QoL. Controlling for these factors in future studies would enhance the validity of the findings.
- Outcome Measures: While the SWAL-QoL provides valuable insights into various aspects of swallowing-related issues, it may not capture all dimensions of patients' experience. Including additional outcome measures or qualitative assessments could provide a more comprehensive understanding of dysphagia's impact.
- Missing Data: The exclusion of patients due to missing data, as mentioned in the study, may introduce bias and affect the robustness of the results. Strategies to minimize missing data, such as ensuring comprehensive data collection procedures, should be considered in future studies.
- Generalizability: The study's findings may not be generalizable to all patients with NMDs, as their spectrum varies widely in terms of severity, progression, and associated symptoms. Replication in diverse patient populations would strengthen the external validity of the findings.

In conclusion, oropharyngeal dysphagia emerges as a prevalent symptom among patients with rare NMDs, with a noteworthy prevalence rate of 52.4%. The onset of swallowing difficulties often manifests insidiously and significantly impacts patients' QoL across various domains. Particularly, subdomains such as burden, eating desire, eating duration, food selection, communication, fear, mental health, social functioning, and symptom frequency of dysphagia were prominently affected in our cohort.

Our findings underscore the significant negative correlation between the EAT-10 scores and SWAL-QoL subscales, highlighting the profound adverse effects of dysphagia on patients' QoL related to swallowing.

These insights emphasize the critical importance of early recognition, comprehensive assessment, and tailored interventions to address oropharyngeal dysphagia in patients with rare NMDs. By acknowledging and addressing the challenges posed by this condition, healthcare providers can strive to enhance the overall wellbeing and QoL of affected individuals, thereby improving overall outcomes and experiences.
